# Microbial community composition of food waste before anaerobic digestion

**DOI:** 10.1038/s41598-023-39991-w

**Published:** 2023-08-05

**Authors:** Linjie Tang, Jack O’Dwyer, Önder Kimyon, Michael J. Manefield

**Affiliations:** 1 School of Civil and Environmental Engineering, UNSW Sydney, Sydney, NSW 2052 Australia; 2 School of Chemical Engineering, UNSW Sydney, Sydney, NSW 2052 Australia

**Keywords:** Biotechnology, Microbiology, Molecular biology, Systems biology, Biogeochemistry, Environmental sciences, Engineering

## Abstract

Anaerobic digestion is widely used to process and recover value from food waste. Commercial food waste anaerobic digestion facilities seek improvements in process efficiency to enable higher throughput. There is limited information on the composition of microbial communities in food waste prior to digestion, limiting rational exploitation of the catalytic potential of microorganisms in pretreatment processes. To address this knowledge gap, bacterial and fungal communities in food waste samples from a commercial anaerobic digestion facility were characterised over 3 months. The abundance of 16S rRNA bacterial genes was approximately five orders of magnitude higher than the abundance of the fungal intergenic spacer (ITS) sequence, suggesting the numerical dominance of bacteria over fungi in food waste before anaerobic digestion. Evidence for the mass proliferation of bacteria in food waste during storage prior to anaerobic digestion is presented. The composition of the bacterial community shows variation over time, but lineages within the Lactobacillaceae family are consistently dominant. Nitrogen content and pH are correlated to community variation. These findings form a foundation for understanding the microbial ecology of food waste and provide opportunities to further improve the throughput of anaerobic digestion.

In 2017, 2 billion tonnes of municipal solid waste was generated globally. In which 84% were collected, and only 15% was recycled^1^. Approximately 60% of this waste stream is organic^2^ and can be anaerobically digested for energy recovery. In the 2018 Australian Government Waste Generation Report, 87% of food waste was landfilled, creating landfill gas and leachate problems. Only 1% of food waste went to energy recovery facilities^3^. The mismanagement of the organic fraction of municipal waste can cause generation of greenhouse gases, landfill leachate, and other harmful products from the uncontrolled decomposition of organic waste^4, 5^. Landfill gas and leachate are harmful to the environment and raise safety concerns^6, 7^. Engineered anaerobic digestion (AD) of organic waste can relieve pressure from landfills by harvesting biogas and nutrients from organic waste. This study focuses on the food waste fraction of the organic waste stream.

Anaerobic digestion relies on microorganisms that decompose organic substances in the absence of oxygen^8^. The digestion process involves four stages (hydrolysis, acidogenesis, acetogenesis and methanogenesis) each carried out by different groups of microorganisms. Complex food matrices are hydrolysed extracellularly into simpler compounds, then acidified and acetified, which then ultimately fermented to acetate, carbon dioxide and dihydrogen by bacteria^9^. These products then serve as substrates for the production of methane by methanogenic archaea^10^. Commercial anaerobic digestion systems have been optimised over decades, focusing on higher biogas yields, higher methane:carbon dioxide ratios, and lower residual solids yields^11^. Rarely has attention been paid to increased digestor throughput, despite the economic trade-off an increased loading capacity can offer^12^. This is surprising given that most of the revenue for AD facilities comes from payments for food waste disposal. Therefore, increasing the loading rate of the digestor improves the financial viability of such facilities and diverts more organic waste from landfills.

Food has an associated microbial community and is highly susceptible to abiotic decomposition and biodegradation. Perish starts as soon as food is harvested, processed, or produced. Anaerobic digestion facilities receive food waste at an early stage of decay from an increasingly active indigenous microbial community^13^. Despite the potential of the microbial community indigenous to food waste to play a role in downstream anaerobic digestion, there is limited data available on the microbial community of food waste feedstock (food waste prior to anaerobic digestion) for AD facilities. For example, the diversity and evenness of bacterial and fungal communities in food waste and how the composition of the microbial community is impacted by environmental parameters such as pH, water content, and element content are unknown. Given that the composition of food waste can vary, it is reasonable to expect that the composition of the microbial community also varies, although this has never been investigated.

This study investigated food waste samples received and pulped by a commercial Australian food waste anaerobic digestion facility. Samples from two stages of basic pretreatment were analysed for microbial community variations and physico-chemical characteristics including pH and element content. Sequencing and quantification of 16S rRNA and ITS showed the structure of and shifts in food waste microbial communites prior to anaerobic digestion. Changes in microbial community composition over the 3 month sampling period were linked to physico-chemical parameters, specifically pH and nitrogen content.

## Materials and methods 

### Feedstock for the food waste digestor

**Figure 1:**
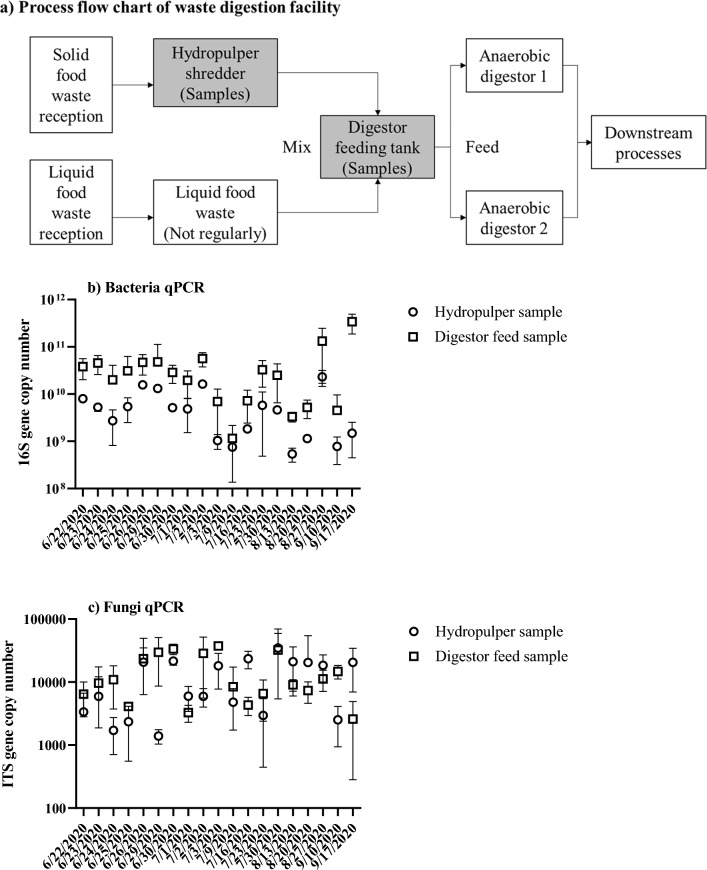
(**a**) Basic process flow chart of the food waste digestion facility. The sampling points are shaded in grey at the hydropulper and digestor feed tank. Liquid food waste includes mainly grease traps and spadable liquid waste. The abundance of (**b**) bacteria and (**c**) fungi per gram of sample (wet weight) taken from the hydropulper (triangles) and digestor feed (squares) was determined by quantitative PCR targeting 16S rRNA gene copies for bacteria and ITS copies for fungi. Gene copy counts represent cell concentration measurements, thus are relative to the wet weight of the sample, and are the average number of technical triplicates.

EarthPower, Sydney, NSW, Australia, provided food waste feedstock. The facility receives solid and liquid food waste from food production facilities, supermarkets, and restaurants. Food waste is sorted and pulped upon receipt. Liquid food waste is mainly oil trap waste and spadable sludge with inconsistent receival rate. The liquid stream is mixed with the pulped solid waste before anaerobic digestion. Two types of feedstock were sampled, hydropulper shredder and digestor feed. Hydropulper samples are pulped fresh food waste with water added in a 3:1 ratio (waste/water), excluding plastic packaging and other undesirable materials. Digestor feeding tank samples include both food waste pulp and liquid food waste. Figure 1a shows a basic process flow diagram of the facility. Samples were collected on 38 dates between 22 June 2020 and 17 September 2020, then stored at − 20 °C individually before processing. EarthPower provided on site water content and pH data for the samples. Non-consistant sampling frequency was decided by EarthPower at their operational convenience. There are approximately 16–20 h of detention time between food waste pulp and digestor feeding tank. Food waste delivered to the facility is processed in the hydropulper shredder on a daily basis. Processed food waste is then stored for 2–3 days in the digester feed tank. Both facilities are operated at ambient temperature (8.7–24 °C, average 12.9 °C for the sampling period).

### DNA extraction and PCR

Genomic DNA was extracted from 0.2 g of food waste samples (wet weight) using the QIAamp PowerFecal Pro DNA extraction kit (Qiagen). Manufacturer protocols were followed, except DNA-free PCR grade water (Sigma) was used instead of the C6 solution for final preservation. Genomic DNA concentration was measured using a Qubit 2.0 fluorometer. Polymerase chain reactions (PCR) were performed on the extracted genomic DNA. The primers used were 1048F (5′-GTGSTGCAYGGYTGTCGTCA-3′) and 1294R (5′-GCCTACGATCTGAACTGAGC 3′) for bacteria, and ITS1 (5′-TCCGTAGGTGAACCTGCGG-3′) & ITS4 (5′-TCCTCCGC TTATTGATA TGC-3′) for fungi. The thermal cycle setup of bacteria PCR is initial denaturation at 95 °C for 5 min, then followed by 30 cycles of 30 s of denaturation at 94  °C, 30 s of annealing at 58  °C, and 40 s of extension at 72 °C and the final extension period is 10 min at 72  °C. The fungus PCR setting begins with an initial denaturation at 94  °C for 5 min, then followed by 30 cycles of 45 s of denaturation at 94  °C, 30 s of annealing at 60  °C, and 45 s of extension at 72  °C and the final extension period is 10 min at 72  °C. Gel electrophoresis was used to identify the integrity of the PCR product with 1% agarose gel run at 90 V for 30 min.

Quantitative PCR (qPCR) was used to quantify bacteria and fungi in food waste samples with a Bio-Rad CFX qPCR machine. The primers used for qPCR are 1048F (5′-GTGSTGCA YGGYTGTCGTCA-3′) and 1194R (5′-ACGTCATCCCCACCTTCC-3′) for the total copy count of the 16S rRNA gene representing the bacteria community^14^ and ITS1 (5′-TCCGTAGGTGAACCTGCGG-3′) and 4 (5′-TCCTCCGCTTATTGATATGC-3′) for the total copy count representing the fungi community. The working solution mix contained 5 µL of SsoFast EvaGreen Supermix (BIO-RAD), 0.1 µL of each primer, 0.1 µL of BSA (20 mg/mL) and 2.7 µL of molecular water. When plating, 96 well plates with 8 µL of working solution and 2 µL of sample were used in each well. The total bacterial qPCR protocol was 3 min of denaturation at 98  °C, forty cycles of DNA segment replication at 90  °C for 20 s, and 62  °C for 50 s. The temperature decreased to 60  °C and was maintained for 10 s for fluorescent reads, following a temperature increase of 60 to 90  °C in 0.5  °C intervals for melt curve analysis. Fungi quantification qPCR protocol was 2 min denaturation at 95  °C, forty cycles of DNA segment replication at 90  °C for 20 s, 55  °C for 30 s, and 72  °C for 60 s. The temperature stays at 72  °C for 10 min^15, 16^. The average efficiency of the qPCR of the 16S rRNA gene and ITS gene copies was 95% and 96%, with an average slope of (− 3.42) and (− 3.46) and an average R2 equal to 0.993 and 0.995, respectively.

### DNA sequencing

Illumina sequencing was used to identify species of bacteria and fungi. The sequencing platform was MiSeq v2 2 × 250 bp. The Ramaciotti Center at UNSW provided the sequencing service, including library preparation and sequencing runs. The bacteria assays were prepared with the 16S rRNA gene amplicon library and the fungi assays with the ITS2 amplicon library (UNSW, Sydney, Australia). Paired-end sequencing results were analysed using QIIME 2 2019.7 pipeline^17^. The sampling depth was set to 1600 for quality control. The Dada2 plugin^18^ was used to denoise the data with trimming length determined using FastQC (https://www.bioinformatics.babraham.Ac.uk/projects/fastqc) for quality assurance. Taxonomy was assigned using the q2-feature classifier^19^ classify-sklearn naïve Bayes taxonomy classifier on Silva 138 release^20^ for bacteria and UNITE 8.0^21^ for fungi taxonomy classification. The reference sequence was modified to increase accuracy to exclude primer overhang. The reference sequence was not cut with the advice of the QIIME2 team^22^. The genomic sequence entries were then BLASTed (https://blast.ncbi.nlm.nih.gov) to acquire species information. Sequence reads extracted from the Illumina results were aligned using MUSCLE. A maximum likelihood phylogenetic tree was generated using MEGA-7^23^.

### Elemental analysis for C, N, and S content

The carbon, nitrogen, and sulphur content of freeze-dried food waste (every other sample) were analysed with a varioMARCO cube at the Solid State & Elemental Analysis Unit XRF at the UNSW Mark Wainwright Analytical Centre (UNSW Sydney, Australia). The manufacturer’s suggested operational protocol was followed^24^.

### Data analysis

Within-sample diversity is represented by Margalef richness^25^ and Simpson evenness^26^ categorized based on sample type, pH, and N%. The data were calculated with Qiime 2 pipeline. The diversity of bacteria and fungi samples were presented in weighted Unifrac PcoA graphs^27^ ategorized by sample type, pH, and nitrogen element content. The bacteriological taxonomy data were filtered to exclude chloroplast sequences. The relative abundance of OTUs is considered the reference abundance of microorganisms in the samples. Due to countless substrains that are low in abundance (< 1%) or only present in a few samples, the top four bacteria species (making up > 40% of community) were kept for further correlation calculations. Similarly, the analysis of the community of fungi included the four most abundant species. GraphPad Prism 9 was used to analyse the correlation between environmental parameters and variances in community structure. Correlations are examined using nonparametric Spearman correlations to cover more than linear relations and to generate heat maps. The P values were calculated with the student t-test in Prism 9.

### Bacterial growth modelling

Bacterial growth in the digestor feed tank was modelled using the Monod equation and key reaction kinetic parameters (Figure S1). The equations reflect the specific growth rate of bacteria ($\mu$max), which depends on the temperature, pH, and nutrient level of the medium. The chosen ($\mu$max) used in the model was conservative since the aim of the model was to determine if there was enough residence time (steady state) for bacterial growth to occur between the two sampling points. Other kinetic parameters used include the biomass yield (Yx/s) and the saturation constant (Ks). Growth kinetics were based on *Lactobacillus,* which represents more than 70% of the bacterial community.

### Ethical approval

This article does not contain any studies with human participants performed by any of the authors.

## Results 

### The abundance of bacteria and fungi in food waste feedstock

To investigate the variation in the microbial communities of food waste, samples were taken from the hydropulper shredder and digestor feeding tank (Fig. 1a) approximately weekly for 3 months from winter to spring. DNA was extracted and the total abundance of 16S rRNA (bacteria) and ITS gene sequences (fungi) was determined using quantitative PCR (Fig. 1b,c). Archaeal relative abundance was below 1%. The 16S rRNA gene copy abundance was 4–6 orders of magnitude higher than the abundance of ITS sequences, indicating that bacteria are numerically dominant. The abundance of fungi was similar in the hydropulper and digestor feed samples (average 4.3 ± 2.0 × 10^4^ and 4.4 ± 2.0 × 10^4^ copies/g, respectively). Bacterial abundance in the digestor feed (average 1.6 ± 2.4 × 10^10^ copies/g) was 26-fold higher than in the hydropulper (average 6.2 ± 6.4 × 10^8^ copies/g) indicating proliferation.

To estimate the growth rate and threshold of bacteria in food waste, a bacterial growth model was developed based on growth rates of *Lactobacillus* (> 70% relative abundance of bacteria community, Fig. 3a,b) under comparable conditions and estimates of available growth substrates (Supplement information 1). The model was used to determine whether the residence time between the hydropulper and the digestor feed tank (~ 16 h) was sufficient to account for the observed 26-fold increase in bacterial abundance. From a starting point of 6.2 × 10^8^ copies/g, the model reached a 16S rRNA gene copy density of 1 × 10^10^ copies/g after 16 h and plateaued at 1.3 × 10^10^ copies/g after 18 h. Growth model data and qPCR data are consistent with the proliferation of bacteria in food waste between the hydropulper and the anaerobic digestor feeding tank.

### Composition of the fungal community in food waste

**Figure 2:**
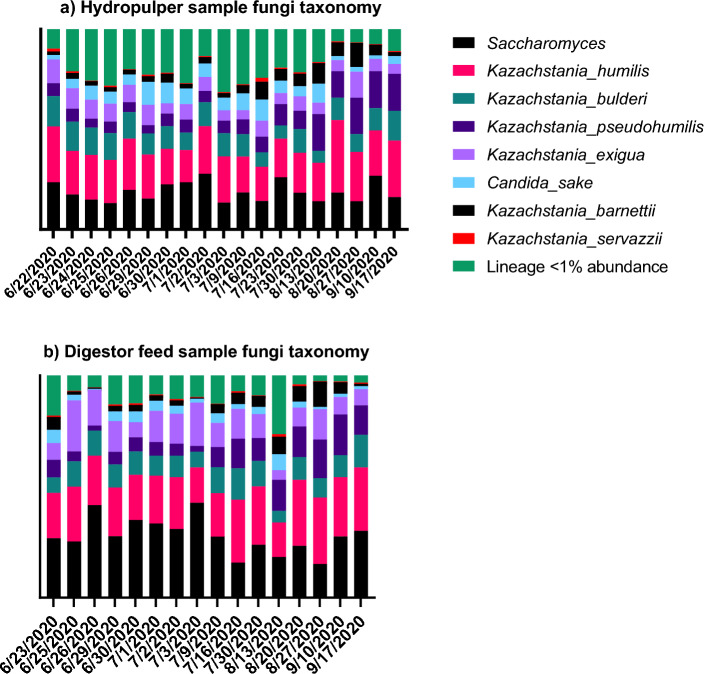
Composition of the fungal community in (**a**) hydropulper and (**b**) digestor feed samples.

Figure 2 shows the structure variation of the fungal community in the hydropulper and digestor feed samples. Sequencing was done on the Illumina ITS platform, and QIIME 2 pipelines were used to process reads. The most abundant fungal lineages belong to the genera *Saccharomyces* and *Kazachstania.* The digestor feeding tank samples held more *Saccharomyces* and the < 1% abundance fungi strains decreased in relative abundance. The composition of the fungal community in the feedstock of food waste was relatively consistent over the 3 month sampling period. The consistent community structure with the lack of proliferation of fungi between the hydropulper and digestor feed samples indicated limited fungal activity in the food waste.

### Composition of the bacterial community in food waste 

**Figure 3:**
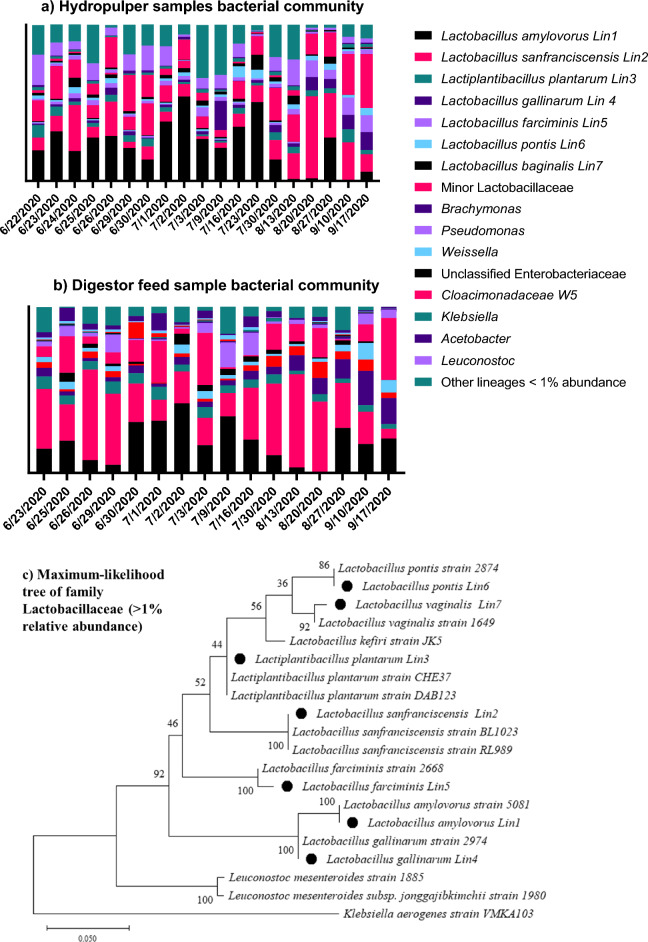
Composition of the bacterial community in food waste samples from (**a**) the hydropulper and (**b**) digestor feed determined with Illumina sequencing. Genus-level identities are presented where possible. Relative abundance excludes chloroplast entries (< 25%). The variation in the abundance of Lactobacillaceae in the bacterial community includes only Lactobacillaceae lineages with > 1% relative abundance (Lin1-7). Minor Lactobacillaceae include all Lactobacillaceae lineages with less than 1% relative abundance. *Lin 1–3* on the graphs correspond to *Lactobacillus/Lactiplantibacillus Lin1-3* in the legend. (**c**) Maximum likelihood phylogenetic tree of Family Lactobacillaceae extracted from the Illumina 16S rRNA Illumina sequence entries (Lin1-7 indicated by solid circle). Number and scale in figure showing the phylogenetic relationship between bacterial lineages observed in food waste and their closest cultured relatives. Numbers represent bootstrap (branch point confidence) values from 500 replicates.

Figure 3a,b shows the variation of the bacterial communities in the hydropulper and digestor feed samples. Illumina 16S rRNA sequencing was used to generate raw reads, and QIIME 2 pipelines were used to process the reads. Lactobacillaceae lineages (including *Lactobacillus* and *Lactiplantibacillus*) dominated throughout the sampling period, with an average total relative abundance in the hydropulper of 64% and 78% in the digestor feed. The relative abundance of Lactobacillaceae was more variable in the hydropulper than in the digestor feed, ranging from 32 to 93%. Sequences within the Lactobacillaceae family and closest relatives from the NCBI database were used to generate a phylogenetic tree to allow species assignation (Fig. 3c). *Leuconostoc* and *Klebsiella* strains were added as outgroups. Three of the most abundant Lactobacillaceae lineages belonged to *Lactobacillus amylovorus* (Lin1), *Lactobacillus sanfranciscensis* (Lin2), and *Lactiplantibacillus plantarum* (Lin3). *Lactobacillus amylovorus* (Lin1) was the most abundant species in the hydropulper, while *Lactobacillus sanfranciscensis* (Lin2) and *Lactiplantibacillus plantarum* (Lin3) were the most abundant in digestor feed samples. Other bacterial lineages above 1% relative abundance include *Klebsiella*, *Leuconostoc*, *Acetobacter*, *Pseudomonas*, *Weissella*, *Brachymonas*, *Cloacimonadaceae W5* and Unclassified Enterobacteriaceae. They were more abundant in hydropulper samples than digestor feed samples. Bacterial lineages with less than 1% relative abundance in the hydropulper had further decreased in relative abundance in the digestor feed.

### Correlations between environmental parameters and community variation

To understand the relationship between food waste physico-chemical characteristics and the composition variations of the microbial community, three characteristics of food waste were measured (Supplementary information). Figure S2a shows the water content and pH variation of the samples over 3 months. Figure S2b shows the proportions of carbon, nitrogen and sulfur in the samples. The digestor feed and hydropulper samples have similar carbon (average of 43.38% and 42.47%, respectively), nitrogen (average of 2.40% and 2.46%, respectively) and sulphur content (average of 0.18% and 0.32%, respectively). Although the sample characteristics between the two sampling points are similar in average, on a daily basis, the sample can be vastly different. Although average elemental contents were similar, the carbon content variation pattern in hydropulper were different from the digestor feed sample from the same day. Similarly, nitrogen content had disagreed abundance variation between same day samples from the two sampling locations.

**Figure 4:**
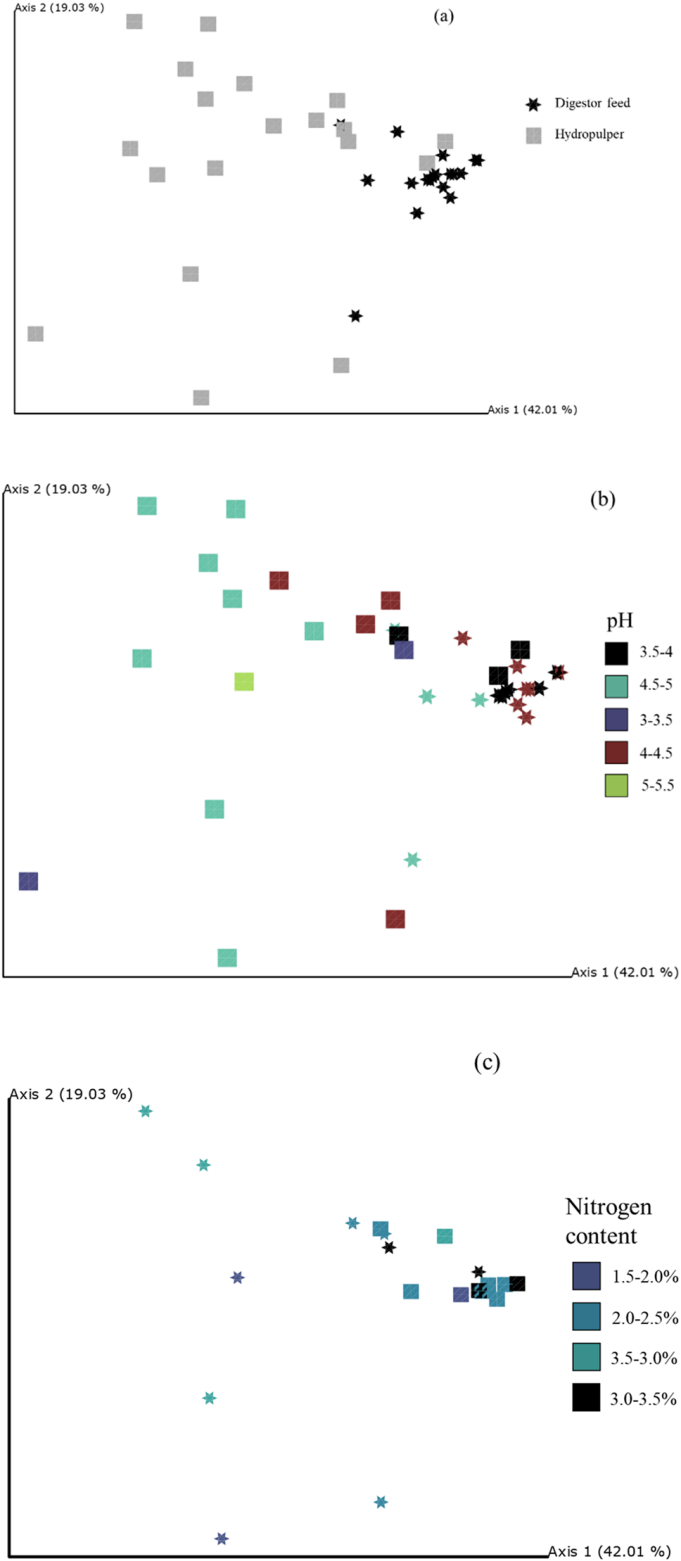
Weighted PCoA graphs show the relationship between environmental parameters and bacterial community composition. Hydropulper (squares) and digestor feed (stars) communities are distinct (**a**). pH (**b**) and nitrogen (**c**) content showed specific grouping at pH 3.5–4 and nitrogen content 3–3.5% (indicated by black color).

Figure 4 shows the bacterial community diversity among samples. The fungal community (not shown) did not return any prominent grouping for all metadata categories. For the bacterial community, two metadata categories, pH and nitrogen element content, were identified with nestings associated with specific ranges. The bacterial community experienced structural shifts from the hydropulper to digestor feeding tank samples. The lower the pH or higher the nitrogen content, the more closely related the bacterial communities were. The pH range of 3.5–4 and the nitrogen element content of 3–3.5% showed a strong influence on the bacteria community.

The diversity within each sample regarding the three metadata criteria, type of sample, pH, and N% of feedstock, are summarised in Table 1. Hydropulper samples had higher bacterial diversity (richness) than digestor feed samples (P = 4.72E−07). When pH was higher than 5, the richness of the bacterial community increased (P = 0.039), and a higher percentage of nitrogen was associated with an increase in bacterial community evenness (P = 0.040).

**Table 1:** Alpha (within-sample) diversity of bacterial and fungal communities, including Margalef richness (a count of different species in the sample, higher means more species) and Simpson evenness (a measure of relative abundance of different species in the sample, higher means less even spread of population among species) indices.

Bacteria	Margalef richness	Simpson evenness	Fungi	Margalef richness	Simpson evenness
Hydropulper average	16.8	0.15	Hydropulper average	15.2	0.15
Digestor feed average	5.9	0.18	Digestor feed average	8.3	0.16
pH			pH		
< 3.5	12.4	0.21	< 3.5	13.2	0.15
3.5–4	5.6	0.18	3.5–4	6	0.17
4–4.5	5.9	0.11	4–4.5	8.2	0.15
4.5–5	14.2	0.16	4.5–5	13	0.15
5–5.5	26.9	0.09	5–5.5	11.1	0.17
N%			N%		
< 1.5	8.2	0.11	< 1.5	8.7	0.16
1.5–2	17.6	0.11	1.5–2	14.2	0.12
2–2.5	6.1	0.15	2–2.5	9.2	0.16
2.5–3	15.2	0.15	2.5–3	12.8	0.14
3–3.5	6.1	0.22	3–3.5	6.5	0.18

**Figure 5:**
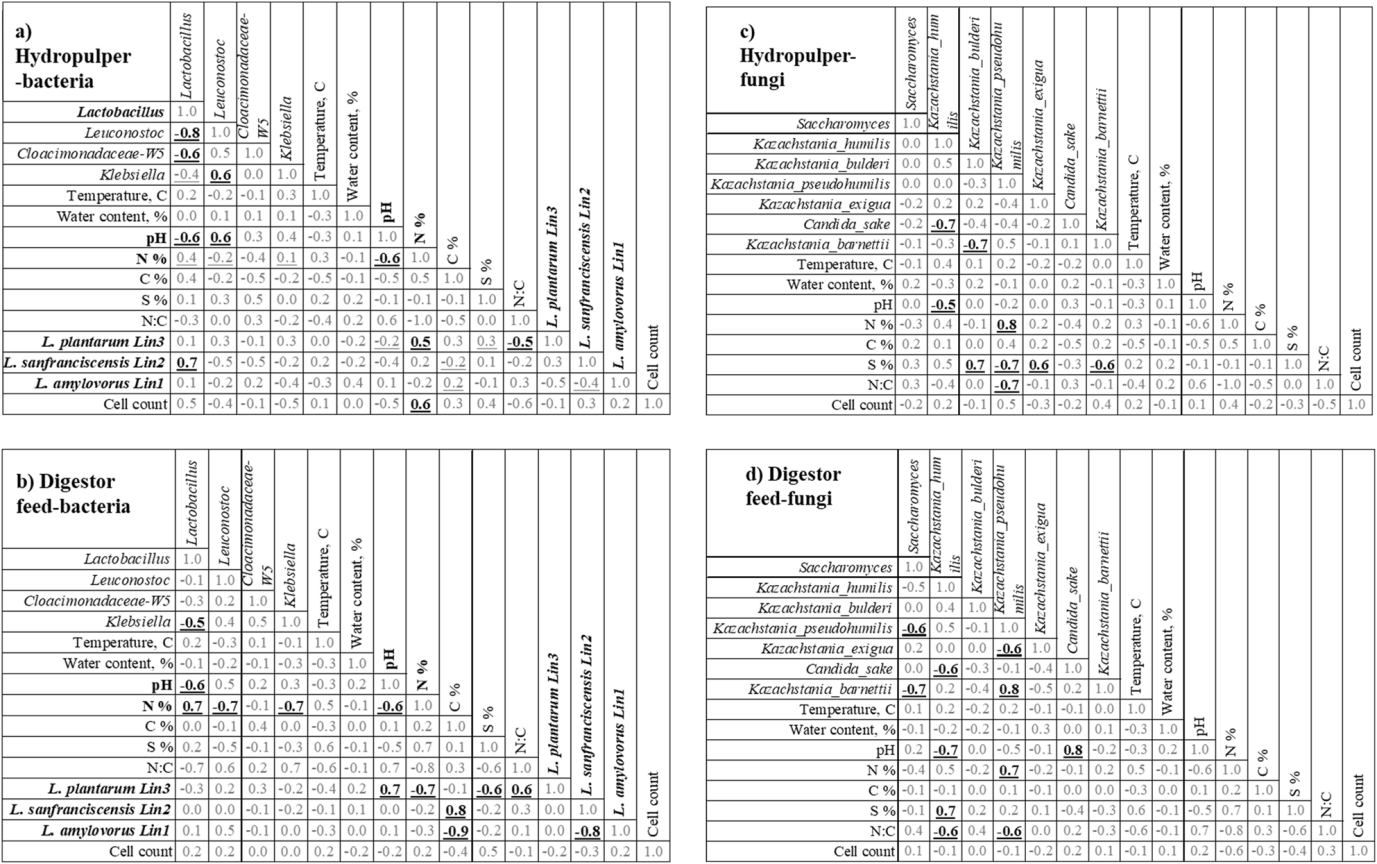
The bacterial heat map indicates the relationships among the environmental parameters (pH, nitrogen content, the variance in relative abundance, and the number of bacterial 16S rRNA gene copies in the sample separated into hydropulper (**a**) and digestor feed (**b**) samples. The negative (dark green) heat map cell indicates a negative nonparametric Spearman correlation and vice versa. The fungal heat map indicates the relationships among environmental parameters, the variance in strain relative abundance, and the number of fungal ITS gene copies in the sample separated into hydropulper (**c**) and digestor feed (**d**) samples. The negative (dark green) heat map cell indicates a negative nonparametric Spearman correlation and vice versa. The underlined entries have a P value less than 0.05 from sigificance test.

Figure 5 illustrates the correlations between environmental parameters, physico-chemical characteristics, abundance species variation, and community variance for both bacterial and fungal communities. Factor scales from − 1 to 1 indicate a total negative or positive nonparametric Spearman correlation. Underlined correlation scores had significant differences (P < 0.05).

Between the hydropulper and digestor feed tank, nitrogen content was positively correlated with abundant bacterial genera (*Lactobacilus*, *Leunocostoc*, and *Klebessila*) and negatively correlated to pH. Furthermore, the BLAST search of Illumina entries allowed for analysing the abundance variations of Lactobacillaceae species under various conditions. The highly abundant *L. plantarum* (Lin3), *L. sanfranciscensis* (Lin2), and *L. amylovorus* (Lin1) were correlated with pH, nitrogen and carbon content with varied dependency.

In the fungal community (Fig. 5c,d), the sulphur and nitrogen element content impacted the hydropulper community, where fungal strains are negatively connected. The pH was not clearly correlated with the relative abundance of fungal strains or the number of ITS gene copies.

## Discussion

Methane yield during anaerobic digestion correlates with the relative abundance of specific bacterial and archaeal lineages in the incumbent microbial community^28^. The composition of microbial communities is determined by the growth and death rates of community members and immigration from outside the system harbouring the community^29^. In the context of anaerobic digestion of food waste, immigration is logically driven by the indigenous community of the foodwaste feedstock. The microbiota associated with feedstock is additionally relevant with respect to chemical transformations it catalyses prior to feedstock entering a digester (biological pretreatment).

Despite its relevance to anaerobic digestion of food waste, relatively little is known about the composition of microbial communities in food waste. One study described the microbial community in food from a canteen from the moment it was discarded and over the ensuing 72 h^13^. Another described changes in community composition of food waste in response to aeration^30^. In addition, a study has explained the variation of food waste composition in response to storage environment^31^. None have described how the community composition of food waste feedstock received by a digestion facility varies over time or within different units prior to entering digesters. The food waste that undergoes anaerobic digestion is mainly composed of bacteria, especially Lactobacillaceae. However, the bacterial community is not stable and varies every day along with the pH and nitrogen levels of the waste. Ammonia, as a form of nitrogen, can inhibit the growth of various species of bacteria and pose selective pressure and shift the microbial community in food waste^32^. This knowledge underpins future attempts to rationally engineer the foodwaste community for improved downstream resource recovery.

In the current study of an industrial anaerobic digestion facility handling food waste, bacteria and fungi were quantified in food waste samples from the hydropulper unit and from the downstream storage unit from which food waste is transferred into the digester. pH ranged from 3 to 5.5 trending downwards throughout the sampling campaign. Water content sat between 80 and 90%. The pH shift of the food waste could result either from changes in the food waste composition as lower pH is associated with carbohydrate-rich food waste and higher pH associated with protein-rich food waste or the storage temperature that affects the production of acidic fermentation products^31^.

Quantification of fungal and bacterial sequences revealed that bacteria are numerically dominant in food waste by several orders of magnitude. This suggests that fungi play a limited role in foodwaste decay or depolymerisation prior to anaerobic digestion. The closely related yeasts *Saccharomyces* and *Khazachstania* were the dominant fungal lineages. *Khazachstania* has been observed previously in food waste and is common in food fermentation communities^33^ *Saccharomyces* are commonly found in food fermentation waste such as the washdown of brewery and bakery factories^34, 35^. The low abundance of moulds may be due to the homogenisation of food waste in handling, transport and ultimately pulping, and the limited availability of oxygen^36^, but the low abundance of fungi generally was suprising. Fungi can be tolerant of low pH and oxygen limitation, so it is possible that the fungi in foodwaste are suppressed through production of antifungal agents by bacteria. The bacteria *Lactobacillus sanfranciscensis* (Lin2) and *Lactiplantibacillus plantarum* (Lin3), observed to dominate the bacterial community in this study, are known to produce antifungal substances^37^. Regardless of the driver of low fungal relative abundance, it appears that the biodegradative abilities of fungi are not being exploited in foodwaste.

The bacterial community was dominated by lineages within the Lactobacillaceae Family (principally *Lactobacillus* species). *Lactobacillus* species are well known for their ability to ferment sugars under oxygen limitation and for the associated release of volatile fatty acids that results in a drop of pH^38^. This has long been exploited in the preservation of foods because lowering the pH below 5 creates conditions unfavourable to most microbes^39, 40^. In essence, *Lactobacillus* species exclude competitors in food waste through pH manipulation. This limits the biodegradation ability of the microbial community in foodwaste to degradative enzymatic activity generated by *Lactobacillus*. Regarding extracellular enzyme activity, abundant *L. amylovorus*, *L. sanfranciscensis,* and *L. plantarum* lineages, are known to produce amylase^41–44^. *L. amylovorus* and *L. plantarum* are also known to produce lipase and protease^45–47^ though proteolytic activity is moderate^48, 49^. *L. plantarum* also produces extracellular feruloyl esterase^50^, while *L. amylovorus* has only been reported to produce intracellular esterase^51^. Therefore, the three most abundant bacteria observed can produce amylase, lipase, protease, and feruloyl esterase, but they are not known to produce cellulase. They probably play a significant role in starch degradation, but have a limited ability to hydrolyse lignocellulosic biomass. There may be potential in bioaumenting cellulase producing bacteria to increase downstream digestion efficiency.

Proteobacteria were notable in their absence in foodwaste^13, 40, 52^. Proteobacteria are capable of degrading various complex substances and are prevalent in anaerobic digestion systems^53, 54^. This supports the potential for bioaugmenting foodwaste with Proteobacteria or altering conditions for the growth of Proteobacteria to exploit their degradation capabilities including cellulase activity^55^.

Aerobic pretreatment of organic substrates can improve anaerobic digestion^56–58^. This is believed to be a consequence of reducing the concentration of easily digestible substrates that can result in rapid decreases in pH due to VFA production under anaerobic conditions, accelerated oxidation of VFAs and the depolymerisation of relatively recalcitrant biopolymers resulting in more extensive digestion and reduced biosolids^59, 60^. Aerobic pre-treatment can increase digestion stability and efficiency^61–63^. These benefits derived from aeration logically hinge on the composition of microbial communities indigenous to food waste and the catalytic abilities encoded therein^30^.

This study showed the variability of microbial community structure in food waste. The samples showed dominance and growth of the bacterial community but not the fungal community. Lactobacillaceae were dominant, and their activity was mainly influenced by pH and nitrogen content. The results inform the potential for adjusting the community via bioaugmentation or supplying air to achieve higher downstream digestion efficiency. These observations form a foundation from which rational engineering of food waste pretreatment conditions can be developed to increase anaerobic digestion throughput.

### Supplementary Information

Supplementary Information.

## Data Availability

The sequencing results can be found in the NCBI Sequence read archive PRJNA805020. Other datasets generated during and/or analysed during the current study are available from the corresponding author on reasonable request.
