# Supplementary material for: Microbial community composition of food waste before anaerobic digestion
Source: Sci Rep. 2023 Aug 5;13:12703. doi: 10.1038/s41598-023-39991-w (PMC10404229; doi:10.1038/s41598-023-39991-w)
Supplement: Supplementary file 1 — Supplementary Information. [file 41598_2023_39991_MOESM1_ESM.docx]

Microbial community composition of food waste before anaerobic digestion

**---Supplementary materials**

Linjie Tang^1*^, Jack O’Dwyer^2^, Önder Kimyon^1^, Michael J Manefield^1^

^1^School of Civil and Environmental Engineering, UNSW Sydney, NSW 2052, Australia

^2^School of Chemical Engineering, UNSW Sydney, NSW 2052, Australia

*Corresponding author: linjie.tang@unsw.edu.au

**Keywords:** Food waste, anaerobic digestion, bacterial community, fungal community

1. **Bacteria growth modelling**

The change in cell biomass concentration (X) (g/L) (equation 2) is based on the Monod equation. The change in the available substrate (S) (g/L) is based on the change in cell mass (equation 3). The discretized model for a time step delta-t can be described by equations 4 and 5. Note that the output of the model is in cell density (CFU/mL), cell mass concentration is converted to cell density by dividing by the cell weight. The cell growth model is presented in Figure S1.

$$\mu= \frac{{}_{max}S}{K_{s}+S}$$

**Equation 1.** Monod Equation (ref. 1).

$$\frac{dX}{dt}= \mu_{G}X= \frac{{}_{max}S}{K_{s}+S}X$$

**Equation 2.** Mass balance of cell biomass in the digester feed tank (ref. 2).

$$\frac{dS}{dt}= -\frac{\mu_{G}}{YF_{\frac{X}{S}}}= -\frac{{}_{max}S}{K_{s}+S}\times\frac{X}{YF_{\frac{X}{S}}}$$

**Equation 3.** Mass balance of available substrate in the digester feed tank (ref. 2) .

$$X_{t}=X_{t-1}+ \frac{dX_{t-1}}{dt}\times\Delta t$$

**Equation 4.** Discretised model for cell biomass where ∆t is the nominated time step.

$$S_{t}=S_{t-1}+ \frac{dS_{t-1}}{dt}\times\Delta t$$

**Equation 5.** Discretised model for available substrate where ∆t is the nominated time step.

Key reaction parameters:

$\boldsymbol{\mu}$max (Max specific growth rate: 0.25 /hr (ref. 3)

Ks (Saturation constant): 4.8 g/L (ref. 4)

Yx/s (Biomass yield): 0.2 g/g (ref. 5)

Temperature: 20 °C ambient temperature

pH: 4-4.5 measured

C:N ratio: 1:12 measured.

**Figure S1.** Bacterial growth model output – Bacterial cell density over time.

1. **Food waste physio-chemical characteristics**

Figure S2 illustrated the variation of food waste water content, pH, elements content include carbon, nitrogen, and sulphur. The variation hinted the potential impact of variable feedstock can have on the digestion efficiency.

**Figure S2.** (a) Water content and pH of food waste in the hydropulper and digestor feedstock. (b) Proportion of carbon, nitrogen and sulphur elements in the hydropulper and digestor feedstock per mass.

**References**

1. Monod J. THE GROWTH OF BACTERIAL CULTURES. https://doi.org/101146/annurev.mi03100149002103. 2003 Nov 28;3(1):371–94.

2. Liu S. Chapter 11 - How Cells Grow. In: Liu S, editor. Bioprocess Engineering (Second Edition). Elsevier; 2017. p. 629–97.

3. Succi M, Pannella G, Tremonte P, Tipaldi L, Coppola R, Iorizzo M, et al. Sub-optimal pH Preadaptation Improves the Survival of Lactobacillus plantarum Strains and the Malic Acid Consumption in Wine-Like Medium. Front Microbiol [Internet]. 2017;8. Available from: https://www.frontiersin.org/articles/10.3389/fmicb.2017.00470

4. Alvarez MM, Aguirre-Ezkauriatza EJ, Ramírez-Medrano A, Rodríguez-Sánchez Á. Kinetic analysis and mathematical modeling of growth and lactic acid production of Lactobacillus casei var. rhamnosus in milk whey. J Dairy Sci. 2010 Dec 1;93(12):5552–60.

5. Hwang CF, Chen JN, Huang YT, Mao ZY. Biomass production of Lactobacillus plantarum LP02 isolated from infant feces with potential cholesterollowering ability. Afr J Biotechnol. 2013 Sep 27;10(36):7010–20.
